# CT- and ultrasound-characteristics of hepatic lesions in patients with multiple endocrine neoplasia syndrome. A retrospective image review of 25 cases

**DOI:** 10.1371/journal.pone.0212865

**Published:** 2019-02-28

**Authors:** Nassim Fard, Heinz-Peter Schlemmer, Friedhelm Raue, Björn Jobke

**Affiliations:** 1 Department of Radiology, German Cancer Research Center (DKFZ), Heidelberg, Germany; 2 Endocrine Practice Heidelberg, Heidelberg, Germany; 3 Telemedicine Clinic/Unilabs, Barcelona, Spain; Ente Ospedaliero Cantonale, SWITZERLAND

## Abstract

**Introduction:**

Liver metastases from neuroendocrine tumors in multiple endocrine neoplasia syndrome are common (75%) and significantly impairs the prognosis. Characterisation of liver lesions in these patients is challenging, as liver metastases are difficult to differentiate from benign liver lesions such as haemangioma.

**Methods:**

In this study we aimed to characterize the radiological findings of hepatic metastases in MEN patients. The findings of contrast-enhanced CT were considered for the main diagnosis. We retrospectively evaluated 25 patients with MEN-syndrome (10 MEN1/ 15 MEN2) including 11 men and 14 women between 28–62 years of age.

**Results:**

Liver metastases (48%, 12/25) and hemangioma (40%, 10/25) were the most common liver lesions among our patients. The most common primary tumors in our MEN1 and MEN2 patients with liver metastases were of pancreatic neuroendocrine tumor (70%, 7/10) und medullary thyroid carcinoma (100%, 15/15) origin, respectively. CT-characteristics were grouped into three main categories, depending on contrast dynamics. The majority of hepatic metastases (75%, 14/25) are presented as multiple lesions with a slow growth in an average 5 years of follow-up-period. We were able to find a common CT pattern and categorise these for each MEN-syndrome. Hepatic metastases in MEN1 presented commonly a blurred arterial enhancement with a low portal venous enhancement and less frequently a prominent enhancement in the arterial phase, which mimics the classical haemangioma. In MEN2 the liver metastases exhibited disseminated mixed hyper- and hypo-enhanced lesions in CT-scans. Moreover, lesion calcifications are pathognomonic in MEN2. The main limitation of this study is the missing histopathological confirmation in the majority of cases.

**Conclusions:**

In this retrospective imaging study, we were able to categorise and find a common CT pattern for hepatic lesions in patients with MEN-syndrome. In order to differentiate these lesions sufficiently, a combination of a 3-phasic CT-scan with US is required. Other liver specific imaging modalities (MRI, CEUS, SMS-PET/CT) should complement the diagnosis in individual cases.

## Background

Multiple endocrine neoplasia syndromes (MEN-syndrome) are rare complex heredity cancer syndromes (incidence rate in MEN1: 2–20/100,000[1] and MEN2: 1/350,000 [2]) with variable endocrine manifestation. Distant metastases in medullary thyroid carcinoma (MTC) occur in approximately 20% of the cases [3]. Liver is a common site for distant metastases in both type of MEN-syndromes and it causes significant increases in morbidity and mortality rate [4–6]. Liver metastases (LM) are found in about 46%-93% of the patients with neuroendocrine tumors (NETs), including those with MEN1 [6]. Distance metastasis of MTC depends on tumor stage and calcitonin (Ctn) level. Liver metastases are observed in 13% of MTC with a serum Ctn level more than 400pg/ml [7]. These lesions are generally hypervascular and exhibit variable atypical imaging appearances, therefore, the differentiation between LM and other vascular hepatic lesions, especially hemangioma, is challenging [8–10]. State-of-the art 68Ga-DOTA-somatostatin analogue-PET/CT improves specificity in detecting the metastases of NET, especially in liver and lymph nodes; nevertheless this imaging technique is not yet a routine protocol for every follow-up in patients with long-term stable disease in Germany. The indications for new treatments (such as systematic therapy with tyrosine kinase inhibitors) are based on RECIST criteria, which cannot be accurately applied to PET. Moreover, its value in MTC is still disputable, for one due to missing correlations between PET positive findings (sensitivity) and calcitonin levels [11–14].

In this article we review liver lesions in 25 patients with MEN-syndrome. The assessment of focal liver lesion is highly relevant in patients with MEN-syndrome because the initiation of a systemic treatment is largely dependent on the existence of metastases[6]. The rational of this study was to acquire more knowledge about the incidence and features of common lesions in MEN patients and building confidence in imaging professionals to support an early diagnosis of disease progression vs. non-relevant incidental findings, reaching for improved survival in these patients [15].

We focus on the lesional imaging characteristics with contrast-enhanced computer tomography (CE-CT) as well as ultrasound (US) that are currently basic care in the life-long surveillance and generally available modalities in radiology departments. We evaluate the imaging characteristics of liver metastases in order to improve the radiological specificity for the clinical benefit in this rare chronic and systemic condition.

## Material and methods

We retrospectively searched our databases for all cases with MEN-syndrome (type 1 or 2), who received radiological assessment from 2004–2014. The initial research yielded 43 patients. All cases were referred from a single specialised local endocrinological partner institution (FAR) and investigated at the DKFZ.

All the data were derived from central scientific data bank (Wissenschaftliche Datenbank (WdB)) of the German Cancer Research Center with an approval from the Ethics Committee of the Medical Faculty of Heidelberg (Nr: S-316/2013). The DKFZ is a primary research center and written informed consent is regularly obtained from all patients at the time of the examination for prospective or retrospective scientific research and potential publication of their anonymized images. The examinations performed in our study were not experimental and were undertaken in the context of routine clinical practice, further informed consent for this retrospective study was not required according to our ethic commission.

The reports and imaging studies were searched for cases with hepatic lesions. Finally, 25 patients were identified suitable for the further evaluation. In these 25 genetically proven cases of MEN-syndrome (type 1–2) the findings of Contrast-Enhanced Computer Tomography (CE-CT) were considered for the main diagnosis, and the group without CT examination, findings of US were used alternatively for the main diagnosis. Moreover, the findings of MRI and PET (^68^Ga-DOTA-somatostatin analogue-PET/CT) occasionally complemented the diagnosis in the cases without histopathological results. In summary CT, US, MRI and PET (68Ga-DOTA-somatostatin analogue-PET/CT) were available in 80% (20/25), 80% (20/25), 36% (9/25) and 24% (6/25) of the cases, respectively (details in Table 1). Additional data such as tumor-markers, time of surgery, duration of the radiological follow-up, histopathology, and presence of extrahepatic metastases were noted for each individual. In 8/12 cases of liver metastases histological confirmation were available and known at the time of image interpretation. The diagnosis in the other four cases of LM (n = 3 in MEN-2 group and n = 1 in MEN-1 group) were based on the rapid tumor-progression and significant increase in tumor-markers over a ten-year period as well as the radiological appearances of the lesions in CT/US in addition to PET-CT and MRT examinations.

**Table 1:** General information of all patients, CT-characteristics as well as ultrasound-findings in patients with liver metastasis.

NR.	MEN Type	Modalities	Number of hepatic lesions	Hepatic imaging Diagnosis	MEN-manifestation	Extrahepatic metastasis	CT-charactristics of metastatic liver lesions.	Lesional calcification	Ultrasound findings of metastatic liver lesions
(1)	1	CT,PET(CT), US	2	LM	Pancreatic NET, pHPT	Lung	ii	No	Hyperechogenic
(2)	1	CT, PET(CT)	multiple	LM	Pancreatic NET, pHPT, adrenal tumor	-	ii	No	-
(3)	1	CT	1	FNH	Pancreatic NET, pHPT, adrenal tumor	Lymph nodes	-	No	-
(4)	1	CT, PET(CT)	1	LM	Gastrinoma, Zollinger-Ellison syndrome	Lymph nodes	i	No	-
(5)	1	CT, US, MRT	1	Hemangioma	Pituitary microadenoma, pHPT	-	-	No	-
(6)	1	CT, US, MRT	1	Hemangioma	Pancreas tumor, pHPT	-	-	No	-
(7)	1	CT, US, MRT	multiple	Hemangioma	Gastrinoma of duodenum, multiple NET of stomach and pancreas	-	-	No	-
(8)	1	CT, PET(CT), US, MRT	multiple	LM	Insulinoma, prolactinoma	-	ii	No	Isoechogenic/ hypoechogenic
(9)	1	US, MRT	1	FNH	pHPT	-	-	No	-
(10)	1	CT, PET(CT), MRT	multiple	LM	Prolactinoma, Pancreatic tumor, adrenal tumor	-	i/ii	No	-
									
(11)	2	CT, US	1	LM	MTC, adrenal tumour	Lymph nodes	iii	Yes	Hyperechogenic
(12)	2	CT, US	2	Hemangioma	MTC, adrenal pheochromocytoma	Lymph nodes	-	No	-
(13)	2	CT, US, MRT	multiple	LM	MTC, adrenal tumour	Lymph nodes, lung	i / ii / iii	Yes	Hyperechogenic
(14)	2	CT, US	multiple	LM	MTC	Lymph nodes, bone	i / ii / iii	Yes	Hyperechogenic
(15)	2	CT, US, MRT	multiple	LM	MTC, adrenal pheochromocytoma	Lymph nodes	ii / iii	Yes	Hyperechogenic
(16)	2	CT, PET(CT)	multiple	LM	MTC	Lymph nodes, lung	i / ii / iii	Yes	-
(17)	2	CT, US	multiple	LM	MTC, bilateral adrenal pheochromocytoma	Lymph nodes, bone	i / ii / iii	Yes	Hyperechogenic
(18)	2	CT, US, MRT	multiple	LM	MTC, adrenal pheochromocytoma	Lymph nodes, bone	i / ii	No	Isoechogenic/ hypoechogenic
(19)	2	CT, US	1	AVM	MTC, adrenal pheochromocytoma	-	-	No	-
(20)	2	CT, US	1	Hemangioma	MTC, adrenal pheochromocytoma	-	-	Yes	-
(21)	2	CT, US	2	Hemangioma	MTC	Lymph nodes, lung	-	No	-
(22)	2	US	1	Hemangioma	MTC	-	-	No	-
(23)	2	US	2	Hemangioma	MTC, adrenal pheochromocytoma	-	-	No	-
(24)	2	US	1	Hemangioma	MTC	-	-	No	-
(25)	2	US	1	Hemangioma	MTC, pheochromocytoma	Lymph nodes	-	No	-

CT: Computer Tomography, US: Ultrasound, MRT: Magnetic Resonance Imaging, PET: Positron Emission Tomography (68Ga-DOTA-somatostatin analogue PET/CT), LM: liver metastasis, FNH: Focal nodular hyperplasia, NET: neuroendocrine tumour, MTC: medullary thyroid carcinoma, pHTP: primary hyperparathyroidism

### Image analysis

The CE-CT-examination were performed by a second-generation 128-row dual energy CT (Siemens Somatom Definition Flash, Siemens Healthcare Sector, Forchheim, Germany) with intravenous application of nonionic iodinated contrast-medium (Imeron 300, Bracco) via an automated injector with the amount and flow rate adapted for body weight. A fixed delay of 10s was used for the early arterial phase (AP) after the cut off value of 120HU was detected (Bolus tracking technique), field of view was from neck to upper abdomen. A fixed delay of 60s was used for the portal venous phase (PVP) with a field of view from the upper abdomen to the proximal part of the upper leg. The majority of the patients were scanned with a 3-phasic scanning protocol (native phase, AP, PVP) with a section thickness of 0.7mm/0.5mm for MPR and reconstructed at 3mm intervals for diagnostic interpretation.

The abdominal US were obtained by two sonographic systems (Siemens ACUSON Sequoia 512 and z.one Ultrasound Systems) with a curved abdominal transducer.

Consensus reading was performed by two radiologists, one in training and one with over 10 years of experience in abdominal imaging who made the final call in cases of disagreements.

In this observational study the CT scans and US studies were reviewed for the presence of lesions. The number (1, 2, multiple ≥3) and location, in addition their morphologic characteristics in CT and US were noted. The maximum diameter of the lesions on axial images were also noted in every first and last imaging study of each patient in order to calculate the relative growth rate during the follow-up period (d2-d1)/(t2-t1).

In proven cases of LM, the morphologic characteristics of hepatic lesions were grouped into three main categories including: group **i,** group **ii** and group **iii** based on their contrast-enhancement pattern in 3-phasic CT-imaging. The detailed definition of each category is presented in Table 2. The prevalence of each category in all patients as well as in each type of MEN-syndrome was evaluated. Calcification of the liver lesions was also noted separately. Moreover, the scans were evaluated for evidence of lymphadenopathy or extra-hepatic metastases.

**Table 2:** Three main categories describing the characteristics of metastatic liver lesions of neuroendocrine tumors in 3-phasic CT-imaging.

	Native phase	Arterial phase	Portal venous phase
**i**	Isodense	Hyper-enhancement with blurry margins	Mild enhancement/ isodense
**ii**	Hypodense	Marginal enhancement	Hypodense +/- mild marginal enhancement
**iii**	Hyperdense(calcification)	+/- Hyper-enhancement	-

CT: Computer tomography, CEA: Carcinoembryonic antigen, Ctn: Calcitonin

## Results

Twenty-five consecutive cases of genetically proven MEN-syndrome were evaluated in our study including 10 patients with MEN1 and 15 patients with MEN2. The mean age of all patients including 11 (44%) men and 14 (56%) women was 40 years (SD ±12). Moreover, the mean average follow-up-period of all cases was about 5±3 years (range: 6 months-12 years). In 40% of all cases multiple hepatic lesions (≥3 lesions) were observed (MEN1: 5/10 vs. MEN2: 9/15 patients), whereas only 16% had a solitary lesion (MEN1: 5/10 vs. MEN2: 6/15 patients).

LM with a prevalence rate of 48% followed by haemangioma in 40% were the top two diagnoses of liver lesions. Other aetiologies of the liver lesions were FNH and AVM in two and one patient respectively. All cases were also screened for lymphadenopathy in our routine protocol. Indeed hepatic metastases were frequently associated with nodal metastases in 8/12 (67%) cases. In general, about 75% of cases with LM were found to be associated with other non-hepatic metastases (Table 1). LM were found in 50% of patients with MEN1 and 47% of patients with MEN2. Multiple LM were also more common than single lesions in each group separately. In each group of MEN1 and MEN2, only one patient had a single LM, and all the other cases had two or more hepatic metastases (MEN1: 4/5 vs. MEN2: 6/7 patients).

**Fig 1:**
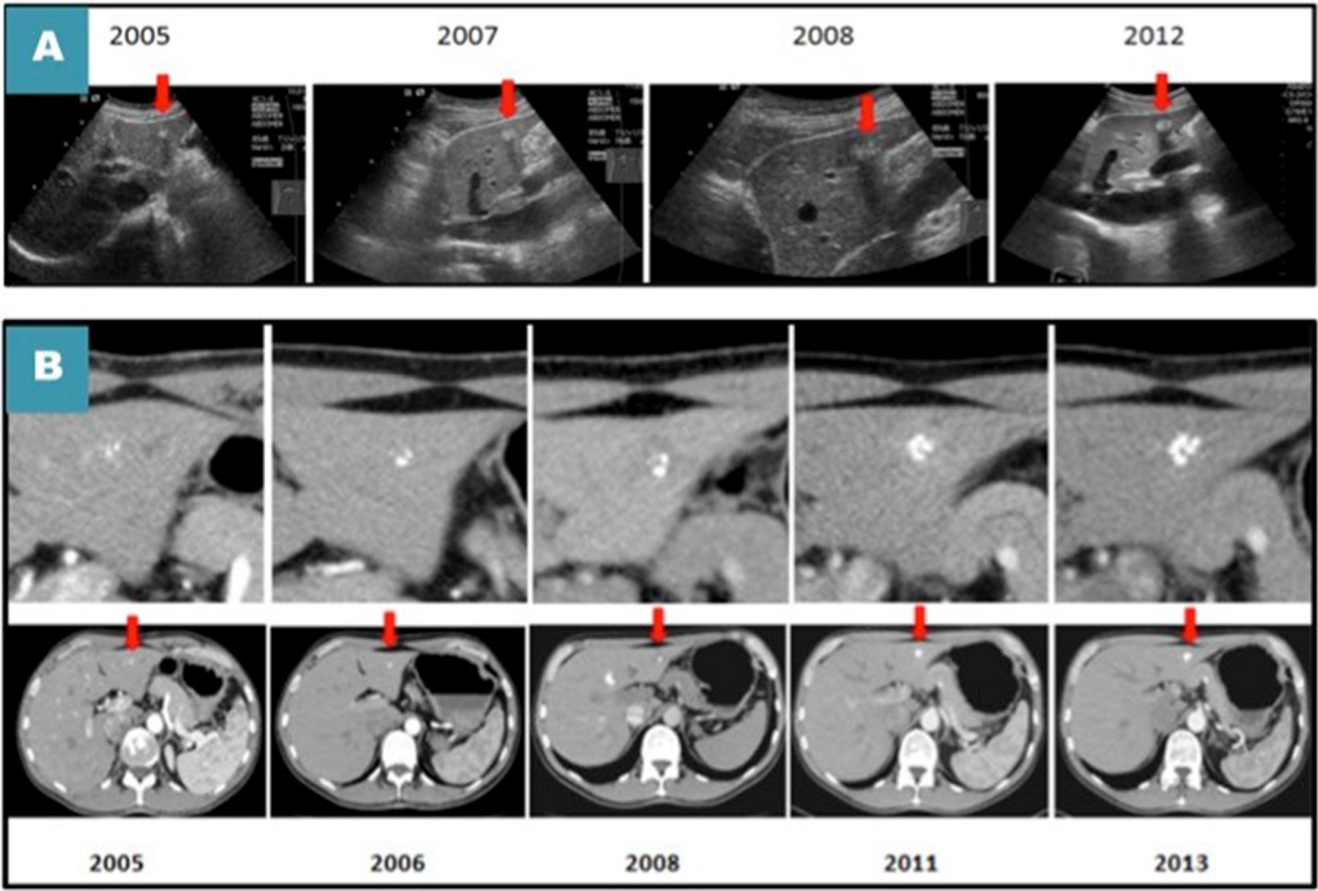
A metastatic lesion in segment 3 of the liver of a single patient in long term follow-up (CT-scan and ultrasound) demonstrating minimal growth over eight years (A) ultrasound (B) CT-scan with zoomed-in calcified lesion.

The vast majority of the metastatic lesions presented a slow growth in the follow-up-period with a mean rate of increase in diameter of 0.8mm/year (range: 0.2 to 2mm/yr, SD:± 0.7). The relative growth rate in MEN1- as well as MEN2-groups were 0.9mm/yr (SD: ± 0.8) and 0.8 mm/yr (SD: ± 0.7), respectively (Fig 1).

**Fig 2:**
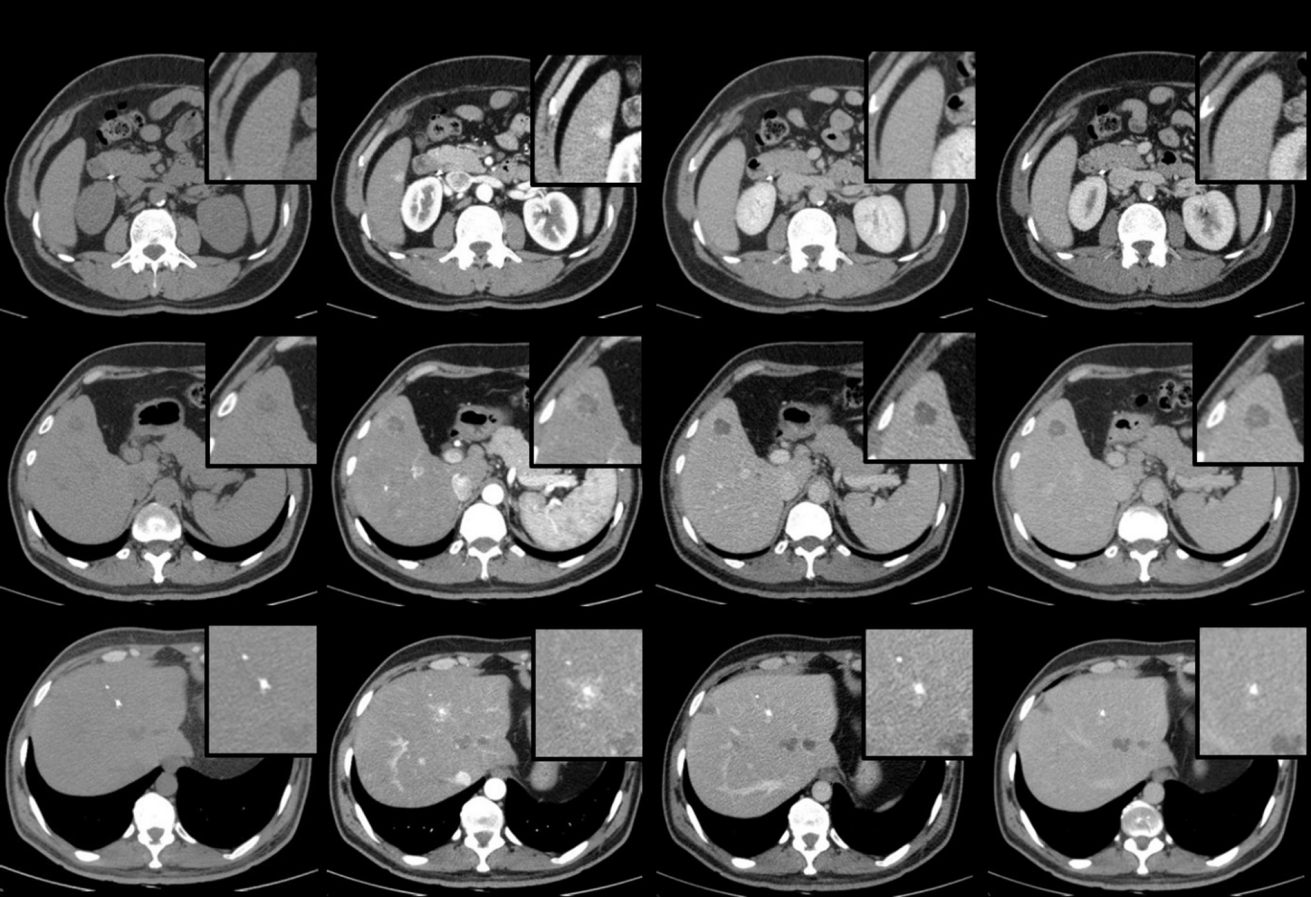
The non-contrast phase, arterial phase, portal venous phase and delayed phase in a 4-phasic MDCT following bolus administration of Intravenous contrast medium in 3 patients with MEN syndrome and pathological proven liver metastasis (arrows). (A) Typical appearance of a hypervascular liver metastasis in segment 6 with avid early contrast enhancement that may wash out or become isodense (and difficult to detect) on more delayed post-contrast images (group i) (B) Hypodense liver metastasis in segment 8 with low-grade contrast enhancement in the arterial phase and rapid wash out in the portal venous phase (group ii) (c) Calcified metastatic lesion in segment 4a with a faint peripheral contrast-enhancement in arterial phase (group iii).

The radiological findings of LM varied from hyper-arterialised lesions to hypodense lesions with low marginal contrast-enhancement (Fig 2). Table 3 presents the prevalence of hepatic calcifications in our cohort.

**Table 3:** The prevalence of liver calcification as well as calcified metastatic lesions MEN1 and MEN2 groups.

Prevalence of calcification	Number of the patient with MEN1 (total = 10)	Number of the patient with MEN2 (total = 15)
Hepatic calcification	0	7/15
LM with calcification	0	6/15

The CT-findings in LM in patients with MEN1 were most frequently central hypodense lesions with marginal contrast-enhancement (4/5 patients). Only in one patient with MEN1 the LM showed an early enhancement in the arterial phase (i).

On the other hand, the most common CT-finding for LM in MEN2 were multiple lesions with a mixed appearance including a combination of all the aforementioned groups (i+ii+iii) (4/7 patients). About 85% (6/7 patients) of these cases revealed an initial disseminated involvement of the liver (miliary pattern). Notably, calcified metastatic lesions were only found in MEN2.

With US nearly all the lesions in MEN1 and all the liver metastases in MEN2 demonstrated a hyper-echogenic appearance (Table 4), which mainly mimics the classic haemangioma. In two cases isoechogenic lesions were found additionally.

**Table 4:** Characteristics of the most common differential diagnoses of hepatic lesions in patients with MEN-syndrome.

	Hemangioma	FNH	Liver metastases MEN1	Liver metastases MEN2
***Radiographic features***	Nodular, homogenous, well defined, internal septa	Well defined with a Central scar	Well-defined	Miliary pattern, multiple
***US***	Hyperechogenic, homogenous, posterior enhancement (less frequent: large lesion with heterogeneous echo pattern)	Varied	homogenous Hyperechogenic, (less frequent: isoechogenic)	homogenous hyperechogenic, (less frequent: isoechogenic)
***CE-CT***	Early peripheral or globular enhancement, persistent centripetal enhancement at venous phase (less frequent: nodular peripheral enhancement, rapid filling, progressive and complete centripetal filling)	Early centrifugal arterial enhancement with a hypodense centrum in venous phase	Low vascular, hypodense lesion with a blurred marginal enhancement (less frequent early enhancement in arterial phase)	Mixed appearance including hyper-vascular lesions with early enhancement in arterial phase as well as hypodense lesions with a blurred marginal enhancement
***Calcifications***	rare	No	rare	Lesional calcification

FNH: Focal nodular hyperplasia, US: Ultrasound, CE-CT: Contrast-enhanced Computer tomography

All patients in MEN2 group underwent a total thyroidectomy after the diagnosis of medullary thyroid carcinoma. The average serum level of Ctn in the patients with MEN2 and LM ranged between 775 picograms per milliliter (pg/ml) to 44276pg/ml (normal range<10pg/mL). Moreover, the serum level of carcinoembryonic antigen (CEA) in the same group at the time of liver metastases ranged between 14 nanograms per milliliter (ng/ml) to 677 ng/ml (normal range<5ng/ml) (Table 5). Patients with a mixed CT pattern including all three types (i+ii+iii) of CT-characteristics of the LM had the highest CEA-level at the time of examination.

**Table 5:** The values of calcitonin as well as CEA at the time of CT-Examination in Patients with MEN2-syndrome.

Number	CT-characteristics	Ctn (pg/ml)	CEA (ng/ml)
1	iii	775	14
2	i/ii/iii	7142	115
3	i/ii/iii	1848	108
4	ii/iii	818	32
5	i/ii/iii	4722	328
6	i/ii/iii	44276	677
7	i/ii	7964	23

In our cohort, haemangiomas presented as single lesions in most of the cases. Multiple haemangiomas were also found in four cases. Only one patient had a calcified haemangioma.

The general patients’ characteristics, CT-imaging features as well as US findings are summarised in Table 1.

## Discussion

MEN1- and MEN2-syndromes may include benign (parathyroid, pituitary) or malignant tumors, which can be secretory or non-secretory, but both syndromes are defined by the presence of NET in two or more different hormonal tissues [16].

LM are common in MEN1- as well as MEN2-syndromes and significantly impair the prognosis in these patients [4–6, 17]. The metastatic hepatic tumors in MEN patients are highly vascular and may be few in number and relatively small (< 1–2 cm) at the time of the primary diagnosis. Early detection of the LM is still a challenge for radiologists as lesions have variable appearances and can be easily missed or mistaken for other benign lesions such as frequent incidental hemangioma [10, 18]. LM in both MEN syndromes may originated from various endocrine tumors, for instance from gastrinoma or thymic carcinoid in MEN1 [4] or primarily from medullary thyroid carcinoma in MEN2-syndrome [19]. In this study we reviewed the spectrum of imaging findings of the LM regarding growth pattern, contrast-behaviour in CT-scans and US characteristics. Due to the rare occurrence of this entity, the recognition and correct interpretation of liver lesions in this syndrome are most likely relatively low and it is our goal to contribute basic information for the understanding of this rare disease.

Nearly half of our patient in both MEN1 and MEN2 groups combined, had hepatic metastases. LM in these patients mostly appeared as two or more (multiple) lesions in the follow up. In both groups LM exhibited a slow growth in an average follow-up-period of 5 years (<1mm/year), which is similar to the slow growing nature of the primary tumors in neuroendocrine heredity syndromes [20]. This feature itself contributes to the difficulty in the differentiation to hemangioma since growth changes may not be detected easily in a prospective follow-up manner. The RECIST-criteria were not applied to our study as the metastatic lesions in the liver were frequently too small to be accurately measured and had no apparent change in size over many years.

Although both MEN1- and MEN2-syndromes are categorised together as familial neuroendocrine neoplasia, they differ in many aspects. In our cohort, LM in each syndrome also depicted different imaging characteristics.

***MEN1*** is a complex syndrome involving endocrine tumors of the parathyroid glands, the pancreatic islet cells, and the anterior pituitary as well as other tumors [1, 16].

In our study the LM in MEN1 patients were mostly poor vascularised lesions with a relatively lower attenuation than liver parenchyma in the PVP(ii). Hyper-vascularised lesions with prominent contrast-enhancement in AP(i) were less frequent in this group. Similar imaging findings were described in review articles previously [15, 21].

**Fig 3:**
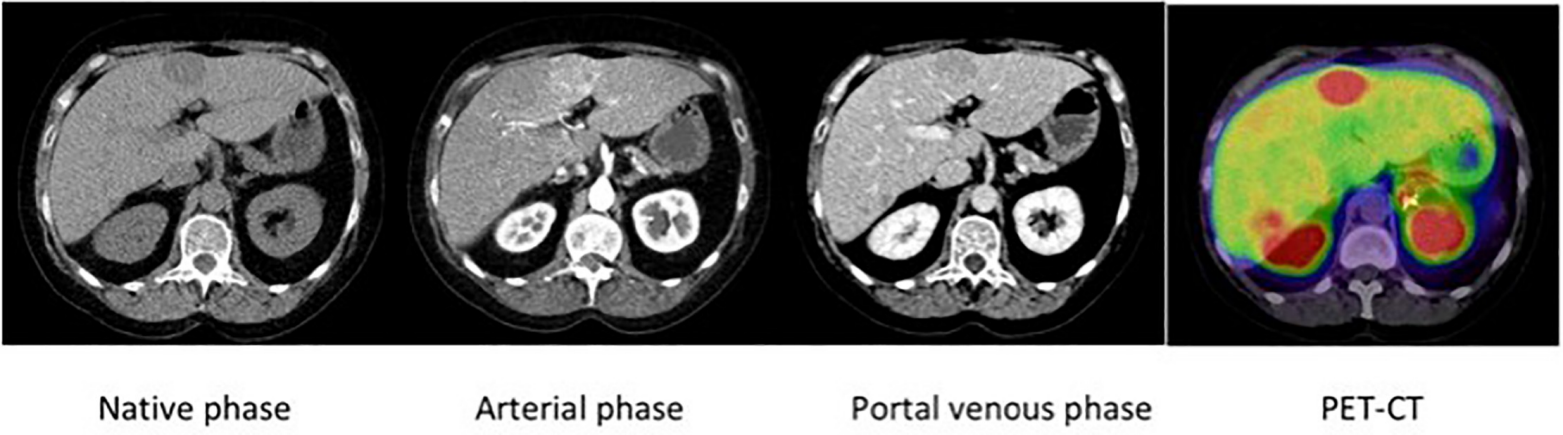
A patient with MEN1 and a liver metastasis. The liver metastasis shows a blurred marginal Enhancement in arterial phase and rapid washout in late phases (group ii) with a prominent uptake in ^68^Ga-DOTA-somatostatin analogue-PET/CT, demonstrating highly homogeneous tumor cell density.

The metastases within group ii manifestation were highly demarcated in the PVP, representing a rapid washout. These lesions were larger in size compared to the lesions in group i and they demonstrated a marginal enhancement during AP. A low contrast-enhancement of a tumoral lesion can result from the central cystic/necrotic changes during the tumor-growth process [22, 23]. However, in our observations there were also lesions with marginal contrast enhancement, but a strong tracer uptake in SMS-PET/CT, representing their homogenous cellular density (Fig 3).

In our MEN1 group, none of the LM were calcified, although, calcifications can be found in larger hepatic metastases in MEN1-syndrome [21].

In the gray scale US the hepatic metastatic lesions were commonly hyperechogenic. Only in one case with fatty liver, the metastatic lesion represented as isoechogenic to low echogenic in comparison to diffused hyperechogenic liver parenchyma. This appearance has been reported previously in the patients with fatty liver. Moreover, tumoral degenerative changes such as central necrosis can also cause a central hypoechogenic appearance in these lesions [21]. In previous studies, variable US-findings are described for LM mainly based on the lesion size. Smaller metastases (<1cm) were reported as low echogenic round shaped lesions, whereas the larger ones (>1 cm) were frequently hyperechogenic with a low echogenic halo. Similar to CT-characteristics, the US findings of these lesions are basically depending on the cellular composition of each tumor.

***MEN2*** is characterised by a combined occurrence of medullary thyroid carcinoma (MTC) with pheochromocytoma and parathyroid tumors [16]. MTC is a well-differentiated neoplasia originating from parafollicular cells (C-cells) of the thyroid gland, which secrete Ctn as well as other polypeptides such as CEA [24].

Similar to findings reported in previous studies, MTC occurred in 100% of our MEN2 patients [2, 25]. Among them, the ones with LM (half of our patients with MEN2) all had highly elevated serum levels of Ctn and CEA, which are known as indicators of distant metastases in MTC [2, 24], as it is reported in the literature that in the presence of increasing tumor infiltration or distant metastases the Ctn-level and CEA-levels will rise to more than 400pg/ml and 100 ng/ml, respectively [19, 26]. Among all patients with LM, the CEA-levels were higher in the patients with mixed type appearance of the LM in comparison to the patients who present one or two different types of the aforementioned CT-characteristics. However, due to the small sub-group size the level of significance could not be evaluated. Furthermore, in the case of stable Ctn-level, elevated CEA level alone can be a sign of dedifferentiation of the tumor which is associated with a worse prognosis and aggressive metastases, although elevated levels of serum Ctn and/ or CEA are not always associated with evidence of tumor-foci during imaging [8]. These cases may have a longer life expectancy with a good quality of life and do not require systemic treatment, though they should be followed by serum tumor-marker measurements and repeated imaging at regular time-intervals, depending on the doubling-time of serum marker level [27].

CT findings of liver metastases in our cohort demonstrated a heterogenic pattern including diffuse multiple hypo-/hyper-enhanced lesion with calcifications (i+ii+iii). Until now, no larger studies have been published exhibiting the imaging features of liver metastases of the familial MTC. Few studies report them as small, hypervascular lesions similar to liver metastases of the NETs [15, 19, 25]. Besides, the literature states that LM of MTC frequently demonstrate a specific initial miliary pattern, which further complicates the radiological detection [25]. We were able to confirm this pattern in nearly 85% of our cases.

Ball et al reported low-attenuating lesions in PVP, which can be easily misdiagnosed with hepatic cysts [19]. Doppman described a doughnut appearance for the liver lesions in MEN2 patients, including a hypervascular rim and avascular centre due to central amyloid accumulation in these tumors [8]. Similar to intra-thyroidal lesions, the metastatic lesions in MEN2 are commonly calcified [28, 29]. We found calcified metastatic lesions in our MEN2 group with a prevalence of 85%. The origin of calcification is yet not clear and it can be either a product of the tumor or result of haemorrhage or necrosis (dystrophic calcifications) induced by chemotherapy [25, 28]. The calcifications are usually scattered and punctuate and can be located both centrally and peripherally in the lesion. Punctuate calcification without a defined mass have also been observed previously and were likewise observed in our study.

The hepatic metastases of MTC are one of the few metastases that tend to be hyperechogenic in US, but they may also be hypoechoic or mixed [25].The most common findings in US of the hepatic metastatic lesions among our MEN2 patients were multiple hyperechogenic lesions, mainly similar to what we found in MEN1 group (Fig 1).The only difference between MEN1 and MEN2 lesions were the presence of calcifications.

### Differential diagnosis

LM in both MEN-syndromes can mimic the appearance of other benign hypervascular lesions especially when they are necrotic or cystic [15]. Benign liver lesions, such as focal nodular hyperplasia (FNH), small hemangiomas as well as other hypervascular metastases are the most common differential diagnosis for enhancing LM in these patients. In Table 5, the most important characteristics of these lesions are summarised.

The hypervascular nature of the hepatic metastases of neuroendocrine tumors causes overlapping radiological findings and complicates the differential diagnosis. There have been a number of cases in the literature in which LM were misdiagnosed as hemangiomas [18, 30, 31]. Hemangiomas are the most common benign non-cystic finding in the liver with features similar to small LM [10, 32, 33]. Similarly, hemangiomas were the second most common finding in our cohort. The high prevalence of these benign lesions increases the rate of radiological misdiagnoses.

### Limitations

Our study was limited by its retrospective study-design and its long time-span. Due to the slow growth nature of MEN, detection and follow-up of the LM are not possible in an appropriate time-period. Another limitation was availability of histopathology results in only 8/12 cases with LM. It should be considered that in most cases, a biopsy was not indicated due to stable tumor-markers. In these cases, the pattern of tumoral progression over time, tumor-markers, CT-characteristics (group i, ii and iii) sonographic appearance as well the findings of ^68^Ga-DOTA-somatostatin analogue-PET/CT and MRT were decisive for the diagnosis. Also, the liver lesions may have originated from different primary tumors depending on manifestation of MEN. Since all tumors in each MEN1-/MEN2-groups exhibit similar imaging characteristics and all histopathologically proven LM confirmed a NET pattern, we believe that this does not taper our results. Moreover, it has been previously shown that the imaging features of NET in a contrast-enhanced CT correlate the histological findings [34, 35]. Another limitation in our study was the variable modalities in the follow-up-controls. This includes application of different imaging modalities as well as different imaging protocols within the same modality. To our knowledge, an optimum radiological monitoring for MEN patients has not been established so far and the available imaging guidelines are tailored to the individual patients and are dependent on local resources and clinical judgments [9, 36]. This diversity was inevitable due to the individual-based follow-up procedures. Moreover, the advances in imaging over the 10-year time period need to be considered. In particular the recent evolving role of PET/CT imaging using ^68^Ga Dota peptide (a somatostatin analog), not only for detecting NET and their metastases in the body, but also for image-guided drug delivery as well as targeted radionuclide therapies [37–39]. Most of our patients did not warrant the application of PET-CT as a first line imaging technique since they presented clinically with stable disease. Other reasons for yet limited application of receptor PET techniques may have resulted from lack of experience with ^68^Ga-DOTA-peptid in the last decade that covered our retrospective analysis, high expenses and lower sensitivity of this technique in detecting small LM with slow growing nature and a low metabolic rate due to higher physiological hepatic uptake of ^68^Ga-DOTA-peptide [40, 41].

The common imaging modalities for follow up controls in MEN-syndrome are US and CT [9] mostly because they are widely available in medical centers. Moreover, their diagnostic role is continually evolving by breakthroughs in new therapies such as Azedra, as these imaging techniques can easily be applied in assessing new sites of disease in patient’s follow-up. Azedra (iobenguane I 131)) is a new FDA-proved drug which targets iobenguane scan-positive, unresectable, locally advanced or metastatic pheochromocytomas and paragangliomas, who require systemic anti-cancer therapy [11]. MRI could not be included in our study since it has only been added in the imaging protocol lately. With regards to MEN-syndrome, a triple-phasic or 4-phasic (native, AP, PVP and late venous) CT-scan is essential to identify LM and a careful inspection is needed. The average MEN patient is relatively young, thus CT radiation dosage over time needs to be considered. US may support the diagnosis, especially applying new techniques such as contrast-enhanced sonography (CEUS), increasing the sensitivity, specificity and detection rate of conventional US [21, 42]. We therefore believe that a second look by other imaging modalities such as CEUS or MRI is extremely helpful for a precise radiological characterization of these oftentimes small liver lesions.

## Conclusion

Metastatic liver lesions in MEN patients are mostly multiple and very slow-growing thus very difficult to characterise in a prospective manner. In this cohort, we categorised our CT-findings in order to provide an index for diagnosing metastatic lesions in liver. LM in patients MEN1 commonly appear with a blurred arterial enhancement with a rapid wash-out in PVP. In MEN2 patients the LM were associated with high levels of Ctn and CEA and exhibited a disseminated mixed appearance including hyper- and hypo-enhanced lesions in CT scans. Lesional calcifications in MEN2 were pathognomonic. With US, a hyperechogenic pattern was the main feature in both syndrome types. There are less common findings in both syndromes, which mimic the classical haemangioma as well as other liver metastases. The essential key for differentiating LM in MEN syndrome from other focal hepatic lesions is a combined radiological examination including US, observation of contrast-behaviour in 3-phasic CT-scan as well as monitoring tumor markers. Furthermore, other liver specific and disease imaging modalities (MRI, CEUS, ^68^Ga-DOTA-somatostatin analogue-PET/CT) can complement the diagnosis in individual cases.

## Supporting information

S1 Data MEN raw data file. (XLSX)

## Abbreviations

MEN: Multiple endocrine neoplasia
US: Ultrasound
MTC: Medullary thyroid carcinoma
LM: Liver metastases
NET: Neuroendocrine tumor
CE-CT: Contrast-enhanced computer tomography
AP: Arterial phase
PVP: portal venous phase
Ctn: Calcitonin
CEA: Carcinoembryonic antigen
CEUS: Contrast-enhanced sonography
SMS-PET/CT: 68Ga-DOTA-somatostatin analogue-PET/CT
